# Ball Milled Graphene Nano Additives for Enhancing Sliding Contact in Vegetable Oil

**DOI:** 10.3390/nano11030610

**Published:** 2021-03-01

**Authors:** Emad Omrani, Arpith Siddaiah, Afsaneh Dorri Moghadam, Uma Garg, Pradeep Rohatgi, Pradeep L. Menezes

**Affiliations:** 1 Department of Materials Science and Engineering, University of Wisconsin-Milwaukee, Milwaukee, WI 53211, USA; eomrani@uwm.edu (E.O.); Afdorri@gmail.com (A.D.M.); 2 Department of Mechanical Engineering, University of Nevada, Reno, NV 89557, USA; asiddaiah@nevada.unr.edu; 3 Department of Physics, University of Wisconsin-Milwaukee, Milwaukee, WI 53211, USA; umagarg@uwm.edu

**Keywords:** graphite nanoplatelet, ball milling, additives, wear, vegetable oil, lubrication

## Abstract

Graphite nanoplatelets (GNPs) as an oil nano additive has gained importance to enhance the lubrication properties of renewable lubricants, such as vegetable oils. Using appropriately processed GNPs is necessary to gain the required tribological advantage. The present study investigated ball-milled GNPs, to understand the effect of GNPs concentration, and applied load on tribological behavior. Pin-on-disk tests were employed, to investigate the tribological performance of the nano-additive oil-based lubricant in the boundary lubrication regime. In order gain an understanding of the lubrication mechanism, Scanning Electron Microscope (SEM), Energy-Dispersive X-ray Spectroscopy (EDX), and Raman Spectroscopy were performed for characterization. The study found that there is a critical concentration of GNPs, below and above which a reduced wear rate is not sustained. It is found that the tribological enhancements at the optimum concentration of GNP in boundary lubrication condition are a result of reduced direct metal–metal contact area at the interface. This phenomenon, along with the reduced shear strength of the ball-milled GNPs, is indicated to reduce the formation of asperity junctions at the interface and enhance tribological properties of the nano-additive oil-based lubricant.

## 1. Introduction

Vegetable oils meet many of the requirements as alternatives to traditional petroleum-based lubricants because they are renewable, biodegradable, non-toxic, and have minimal environmental pollution and production costs [1]. Vegetable oils also exhibit higher lubricity, lower volatility, higher shear stability, higher viscosity index, higher load carrying index, and superior detergency and dispersancy when compared with mineral and synthetic oils [2,3]. Despite the environmental advantages to using pure natural oils, especially canola oil, they do suffer poor oxidative stability, which is substantially determined by the nature of the base oils [4]. They are also susceptible to oxidative degradation due to the presence of free fatty acids and the presence of double bonds in the carbon chain. These shortcomings of canola oils have led to studies looking at enhancing their tribological performance by the use of nano additives [5,6] in addition to chemically modifying them to be usable as mixed lubricant additives in petroleum-based oils [7,8].

Wear mainly occurs in the boundary and mixed lubrication regimes during conditions of transient state (i.e., start-up and shutdown), high load, and low-speed operation [9,10]. In the boundary lubrication regime, direct contact between lubricating surfaces commonly occurs even though a fluid is present. In the mixed lubrication regime, the coefficient of friction is unstable, and the lubricating surfaces are separated by a very thin lubricant film with an average thickness of less than 1 mm [11,12]. Mineral, organic, and synthetic oils are not always efficient enough to satisfy the demands of a high-performance lubricant; therefore, mixing additives with base fluids is an approach to improve the lubrication ability and to reduce friction and wear. The use of additives has a large influence on the performance of lubricants that make it possible to fulfill the increasingly complex demands placed on lubricants. The results of previous studies indicate that nanoparticles used as lubricating oil additive can improve the tribological properties of base oils [13,14,15,16,17], in addition to commercially layered compound powders as solid lubricants usually dispersed in oil [18]. The addition of nanomaterials as additives in base lubricant oil is unique, compared to traditional bulk materials, due to their extremely small size and high specific surface area [19,20,21]. Nanoparticle solid lubricants, such as molybdenum disulfide (MoS_2_) [22] and nano-graphite [16,23] dispersed in oil, exhibited beneficial effects by reducing the friction and wear. The minimum coefficient of friction achieved using MoS_2_ [24] was 0.16 and nano-graphite [25] was 0.08 when used as an oil additive.

Nanoparticles as oil additives have been widely investigated. Nanoparticles used as oil additives can help in reducing the coefficient of friction. The tribological behavior of nano-graphite nanosheets as an additive in paraffin oil was investigated by Huang et al. [26] with a four-ball and a pin-on-disk tribotester. The graphite nanosheets with an average diameter of 500 nm and a thickness of 15 nm were prepared by stir ball milling. The maximum loads for these boundary conditions of the lubricating oil were determined according to the ASTM D2783 standard method. As a lubricant additive in oil, graphite nanosheets demonstrated better tribological properties than in pure paraffin oil, when an appropriate additive concentration was used. The low shear strength between the sliding surfaces resulted from the employment of graphite nanosheets with the layered structure is responsible for the observed improvement on tribological properties.

Lee et al. [23] studied the tribological properties of graphite nanoplatelets (GNPs) as additives in industrial gear oil and found that the nano lubricant containing GNPs had a lower coefficient of friction and less wear. Some experiments showed that the modification of GNPs can improve the dispersion of graphene platelets in base oil, the wear resistance, and the load-carrying capacity of the machine, as well as reduce the resistance to shear and wear scar diameter [16,17,27]. Lin et al. [28] studied the tribological properties of chemically modified graphene platelets (MGPs) as additives in 350SN base oil and reported that the oil containing only 0.075 wt.% of MGP improved the tribological properties [16]. Eswaraiah et al. [29] studied the tribological properties of graphene-based engine oil and found that, for 0.025 mg/mL of graphene in engine oil, the friction characteristics and wear scar diameter were reduced by 80% and 33%, respectively. However, the tribological properties of these inorganic additives and graphite or GNPs in inorganic industrial oils are acceptable. Su et al. studied the tribological properties of graphite nanoparticles as LB2000 vegetable-based oil additive and reported that the addition of graphite nanoparticles in LB2000 vegetable-based oil made the morphologies of wear scars smoother.

In this study, GNPs were studied as an oil additive through sets of experimental work. Canola oil was chosen as the base oil. The study attempts to explain the enhancement mechanisms of GNPs at the contact surface during tribological testing. Coefficient of friction (COF) and wear rate were studied though pin-on-disk tests. The effect of different concentrations of GNPs on the friction and wear properties of the lubricant was also investigated.

## 2. Materials and Methods

### 2.1. Materials

GNPs were used as nano additives in canola oil. GNPs were ball milled for three hours, to reduce the number of layers. The average thickness of the GNPs (Asbury, Asbury, NJ, USA) was approximately 10 nm, with an average platelet diameter of approximately 5 µm. Graphite layers have the weak van der Waals bonding and strong bonds in the basal plane. As a result, the shear force during milling can potentially reduce the number of layers in GNPs. GNPs are strongly hydrophilic and easy to agglomerate and precipitate in oil. Therefore, this was particularly important to ensure uniform dispersion of particles in the canola oil when GNPs were added to the base oil as an additive. All particles were added (in several additive weight fractions) to the canola oil and stirred with three different methods, to find the optimum mixture methods. One of the important factors for oil and additive performance is to have a well-dispersed mixture such that its composition is considered homogenous and keeps it well-dispersed during the tribology test. The three methods of mixing were (1) shaking for 20 min, at room temperature; (2) using an ultrasonic mixer (UM), for 2 h, at 60 °C; and (3) shaking and then using an ultrasonic mixer, as explained in (1) and (2) [30].

### 2.2. Characterization Techniques

Several characterization methods were employed to study the tribological behavior and wear mechanism of nanolubricants. The worn surfaces were characterized, using Scanning Electron Microscopes (SEM) (Hitachi S-4800 Ultra 157 High Resolution Cold Cathode Field Emission Scanning Electron Microscope), Cu-Kα X-ray diffractometer, Energy Dispersive Spectroscopy detector, and Raman microscopy. X-ray diffraction (XRD) was performed with an X-ray diffractometer (Bruker D8 Diffractometer, Trenton, NJ, USA), using Cu Kα1 radiation (λ = 0.15406 nm). X-ray diffraction spectra were produced in the 2θ range, from 15° to 85°, at a scanning rate of 0.5°/min. Raman spectroscopy was performed, using a Raman microscope (Renishaw Inc., 1000B, San Antonio, CA, USA), to determine the chemistry of tribolayer on the worn surfaces. Spectral patterns were produced, using a Helium neon red laser with a wavelength of 633 nm and power of 8 mW. Three-dimensional surface metrology was performed, using an optical profilometer to calculate the surface roughness and observe the surface topography.

### 2.3. Tribological Performance

The pin-on-disk tests were conducted according to American Society for Testing and Materials (ASTM) G99-17 standards and consisted of 2024 aluminum alloy (T4 heat treated) with an Hardness Rockwell B (HRB) of 73 as the pin material and 440C stainless steel with an Hardness Rockwell C (HRC) of 60 as the disk material. The pins were machined to dimensions of 6 mm in diameter and 10 mm in length, with a hemispherical tip. The disks had dimensions of 55 mm in diameter and 10 mm in thickness. The disks were originally polished to a surface roughness having an arithmetic average, S_a_, of 0.15 ± 0.05 μm. Pin-on-disk tests were conducted at room temperature and ambient conditions, to study the tribological behavior of different nano-additive oil-based lubricant. The pin and disk specimens were cleaned by acetone before, and by hexane after, each test. A thermocouple was also deployed to measure the final temperature of the lubricant. During each of the tests, the surface of the disk was completely immersed by the lubricant, thus continually lubricating the pin/disk interface during the tribology tests. Prior to each experiment, and after each test, the pin and disk samples were cleaned by ultrasonic cleaner with hexane. In order to verify the validity of the results, each test was repeated a minimum of three times, ensuring repeatability and accuracy of the results, and the average of data are reported. The test conditions are detailed in Table 1. The normal load and friction force measurements were monitored for each test, using a two-beam type load cell that read the normal load from a static hanging mass and the friction force from as the tangential force of the pin holder. The linear wear-loss was acquired through a linear variable differential transducer (LVDT), with an encoder, which recorded the vertical displacement of the pin. It should be noted that base lubricants with no additives were also used to isolate the results and investigate the effect of particles explicitly.

## 3. Results and Discussion

### 3.1. GNPs Characterization

GNPs offer a better antifriction effect than a single-layer graphene and graphite [31,32]. The shear force applied on a few layers of graphene between contact surfaces can cause the exfoliation of GNPs [33,34], thereby it can contribute to the lubrication behavior of the base oil. In the case of very few layers of graphene, the continuous exfoliation due to shear forces between the mating surfaces can lead to form several single layer graphene sheets which cover the contact surfaces and consequently reduce metal–metal interaction and contact. One approach to reduce the number of layers and change the structure in GNPs is mechanical milling [35]. During milling, the shear forces intercalate/exfoliate GNPs. The weak van der Waals bonding between GNPs layers and the strong bonds in the basal plane between C-C atoms are part of the nature of GNPs layers. As a result, the shear force during milling can potentially reduce the number of layers in GNPs. Ball-milled GNPs are then dispersed in the canola oil, for the desired volume fraction of GNPs (0.05, 0.10, 0.15, 0.20, 0.25, 0.30, 0.35, and 0.40 vol.%).

**Figure 1:**
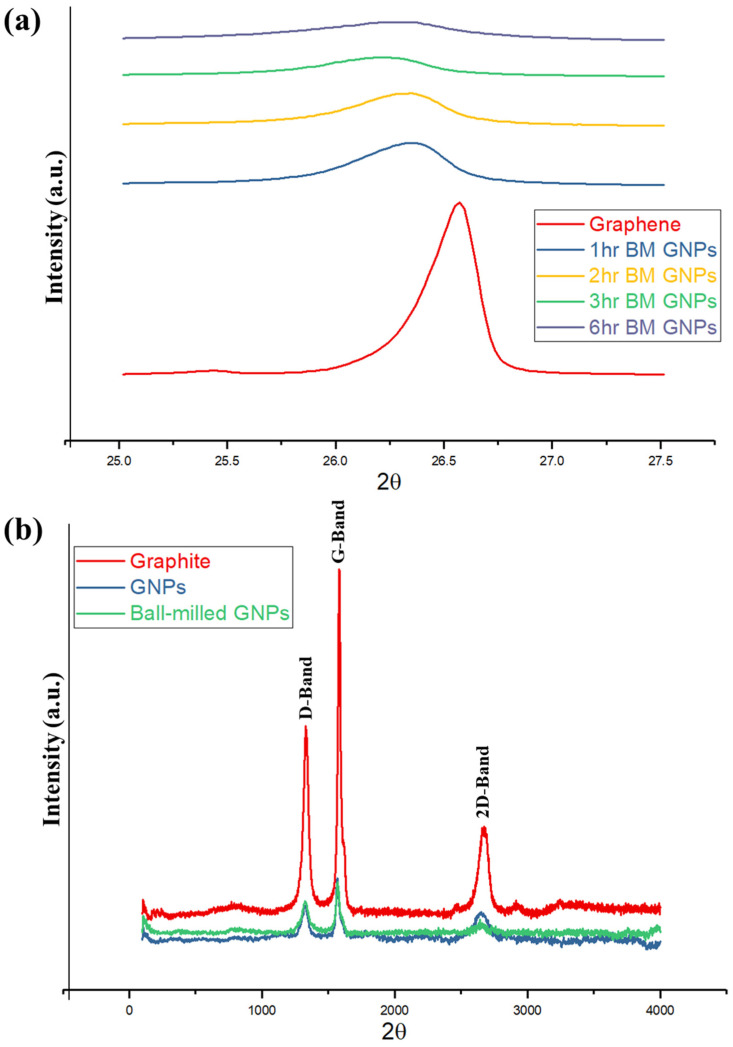
(**a**) X-ray diffraction pattern for graphite nanoplatelets (GNPs) before and after ball milling for 1, 2, 3, and 6 h. (**b**) Raman spectra of graphite, GNP, and ball-milled GNPs tribological properties.

XRD patterns of the GNPs before and after ball milling (for 1, 2, 3, and 6 h of milling time) are illustrated in Figure 1a. The ball-milling process breaks the large crystallites of GNPs and leads to smaller particles; thereby, a significant number of defects appear in the GNPs. A sharp asymmetric (002) peak is observed at angle 2θ = 26.6° for as-received GNPs. After ball milling, the peak broadens and the height of the peak decreases. Broadening of the (002) peak is due to the disorder caused by high-energy ball-milling. The position of (002) basal plane peak also shifts to a lower angle with ball milling, resulting in an increase of interlayer spacing d_002_. The result now provides evidence of the d_002_ spacing increase from 3.34 Å for GNPs without ball milling to 3.37 Å after one-hour ball milling, indicating disorder along c-axis. Assuming that the carbon layers are distributed uniformly, the Scherrer equation is used to find the size of the nanocrystallites. From these XRD results, the size of the crystallites is found to be 27.8 and 21.3 nm before and after 1 h ball milling, respectively. After three hours of ball milling, the peak broadens even further, where d_002_ increases and crystal size decreases. No further change was observed from the XRD patterns for the GNPs samples ball-milled for longer time (over 3 h). The monolayer graphene does not have any (001) peak, since it is two-dimensional [36]. This result has further strengthened the conviction that the reduction in the intensity of (002) peak is attributed to the exfoliation of layers in GNPs along the “c” direction. The results share a number of similarities with Xing et al.’s [37] findings.

Figure 1b shows the Raman spectrum of graphite for as-received GNPs and 3-h ball-milled GNPs. As the nanoplatelets are ball milled, the Full Width at Half Maximum (FWHM) of D the peak increases from 50 to 59 cm^−1^, which is the result of the disorder. The intensity of the 2D band, after ball milling, decreases, and the peak broadens. The decrease in intensity of 2D band is due to the disorder along the “c” axis and is proportional to the crystal size [37]. This result ties well with XRD results wherein the disorder increases after ball-milling of GNPs. A similar conclusion was reached and explained by Kaniyoor et al. [38], where a high amount of defect can suppress the 2D-band and a bump-like region emerges. Since no chemical treatments were performed on the GNPs samples, the peculiar 2D-band shape cannot be due to functional groups, and the defects are the reason for the appearance of the 2D-band shape. This aspect of the research could simply mean that the D-band appears in graphite only in defective/disordered samples or at the edges. Thus, as mentioned earlier, the ball-milling process transformed the large crystallites into small particles and introduced a significant number of defects. 

#### 3.1.1. Tribological Performance

**Figure 2:**
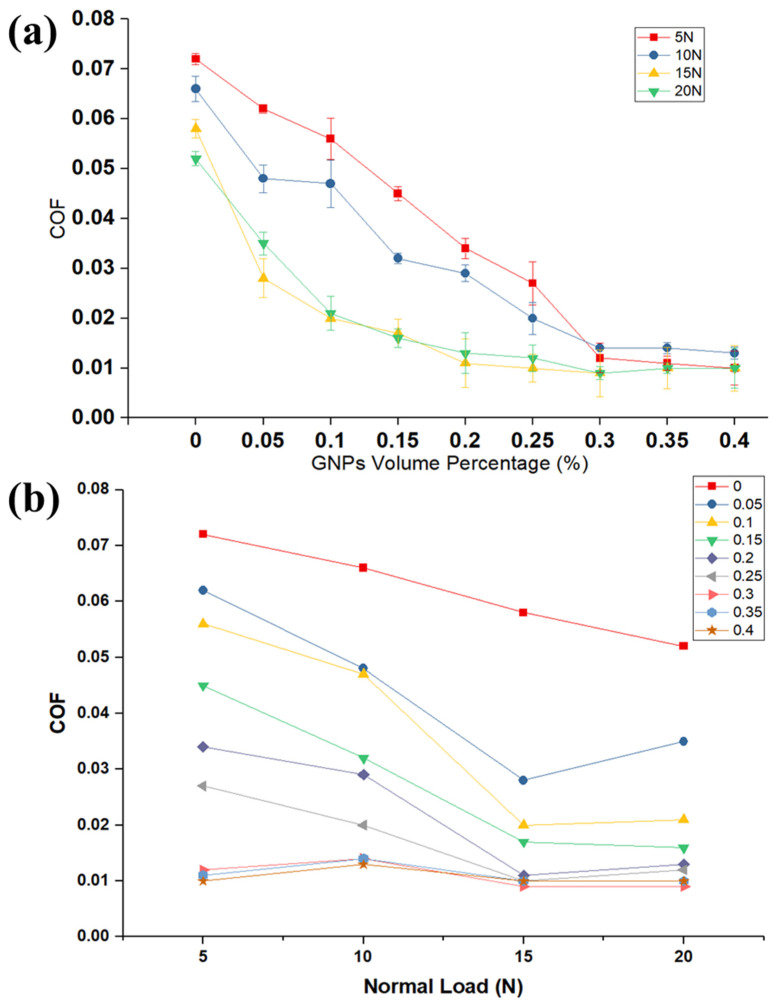
(**a**) Variation of the coefficient of friction with varying GNPs percentage in the oil at different loads. (**b**) The variation of coefficient of friction (COF) with different loads at several volume percentages of GNPs.

To assess the tribological performance of nanolubricants, the pin-on-disk test was employed. Figure 2a shows the correlation between the volume fraction of nanolubricants and COF and compared with neat oil. Our findings on COF demonstrate that the COF decreases by adding GNPs into the oil, because the GNPs can reduce the real contact area between two surfaces. This result highlights the role of GNPs in filling the inter-asperity valleys to improve the tribological performance of the base oil. The GNPs have the ability to easily penetrate the interface and form a continuous tribolayer on the mating surfaces and align themselves parallel to the relative motion. This appears to be a cause for reduction in shearing stress with relative ease, providing adequate lubrication. The most striking result to emerge from the data is that the COF gradually decreases by increasing the volume fraction of GNPs. Interestingly, this correlation is related to the presence of more GNPs in the nanolubricant and between mating surfaces at a high-volume percentage of GNPs and consequently decreasing the direct real contact area between worn surfaces. Moreover, nanolubricants with higher GNPs concentration can deposit thicker tribolayer on the worn surface. The implications of these findings are that the lubricant tribolayer greatly reduces the delamination and failure on the worn surfaces and, thus, decreases the roughness of the surface and direct surface-to-surface contact. This suggests that the corresponding lubrication regime gradually transfers to a good lubrication regime. It is apparent from Figure 2b that the effect of GNPs is more influential to improve the COF at higher loads and GNPs concentrations. As was noted in the Methods section, the experimental tests are such that they lie in the boundary lubrication regime where the lubricant film thickness between surfaces approaches the surface roughness. It was reported in the literature that not only there is considerable contact between tribopairs and their asperities, but also some area of the contact surfaces is totally separated by the lubricant film in boundary lubrication regime and there is no connection between asperities. It is fundamental to note that an increase in the normal load can result in more lubricant beong squeezed out of the contact surface, which consequently decreases the lubricant film thickness between contact surfaces. This would escalate the probability of contact between two surfaces and high friction. However, in the case of the nanolubricant, a higher load increases the probability of GNPs engagement in the contact and more GNPs entrapped between contact surfaces; therefore, the importance of GNPs can be emphasized in the boundary lubrication regime. Taken together, the most surprising finding is that the influence of the high concentration of GNPs to lower the COF is far greater than the low concentration of GNPs in the oil. For example, the presence of the 0.1 and 0.3 vol.% of GNPs at 15 N applied load lowers the COF by 39% and 73% improvement, respectively, when compared to the neat oil. 

**Figure 3:**
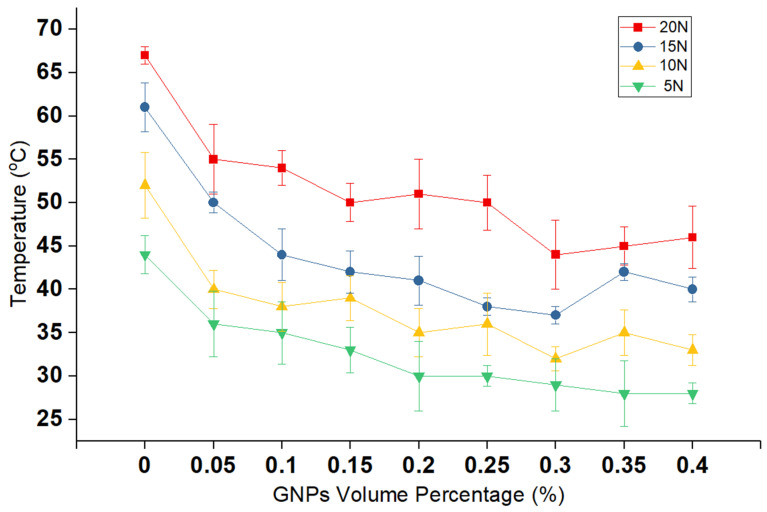
The variation of surface temperature at different volume percentages of GNPs at several loads.

The results on the steady-state temperature of the lubricant at the end of the experiment are compared in Figure 3. In the case of lubricants, a lower friction coefficient results in less heat production, and, consequently, the final temperature is lower. These values correlate satisfactorily with previous researches [39] and further support the concept that the GNPs also have proven to affect the thermal properties of the lubricants, such as conduction, which could result in better heat dissipation. The evidence from Figure 3 points towards the intimates that this effect appears to result in a significant reduction in the temperature, in comparison to the neat lubricant. 

**Figure 4:**
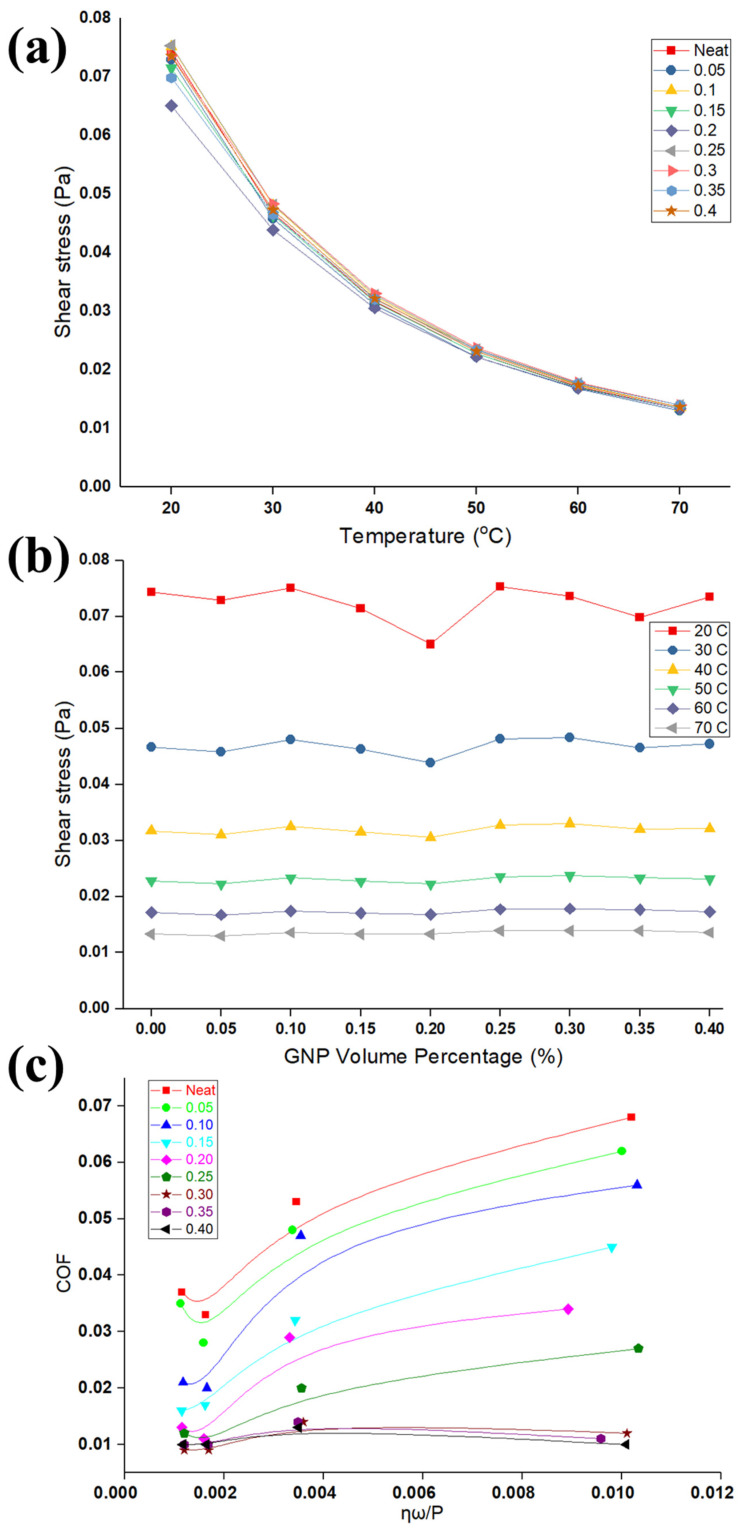
(**a**) Viscosity versus temperature for different concentrations of nanolubricants. (**b**) Viscosity versus volume percentage of GNPs for different loads. (**c**) Stribeck curve for different volume percentage of GNPs.

The viscosity of lubricants is affected in the presence of the GNPs suspended in the oil that results in increasing the dissipated energy and the viscosity of the nanolubricants. Figure 4a,b shows the results of viscometer of neat oil and nanolubricants for different concentrations of GNPs at several temperatures. It is apparent from Figure 4a that the viscosity of nanolubricants significantly reduces with temperature. An explanation of dropping in the viscosity with temperature is due to the inter-molecular and inter-particle adhesion forces that become weak with the increase in temperature; in consequence, it causes a reduction in the viscosity of the nanolubricants. An additional possible reason is increased Brownian diffusion at higher temperatures that can affect the viscosity to decrease it. More recent evidence [40] proposes that the viscosity of nanolubricants increases considerably at a higher volume fraction of particles, while it obviously decreases at a higher temperature.

The increase in viscosity of nanolubricants after the addition of GNPs is insignificant. Figure 4b highlights that viscosity is slightly constant at a certain temperature with adding a different concentration of GNPs. It obtained comprehensive results, proving that these nanolubricants have a linear relationship between viscosity and shear stress. These findings support the notion that nanolubricants behave as a Newtonian fluid, where viscosity remains constant, no matter the shear applied load, at a constant temperature. The reason for this behavior remains unclear, as most of the existing explanations seem to be speculative [41,42]. However, Heine et al. [43] investigated through the molecular dynamics simulations of equilibrium structure and the response to imposed shear on suspensions of spheres, rods, plates, and jacks, that the rod and plate systems show noticeable particle alignment, which helps to minimize the frequency of particle collisions. Similarly, it is expected that the GNPs having sheet structure could align themselves along the shear direction; this requires further investigation.

**Figure 5:**
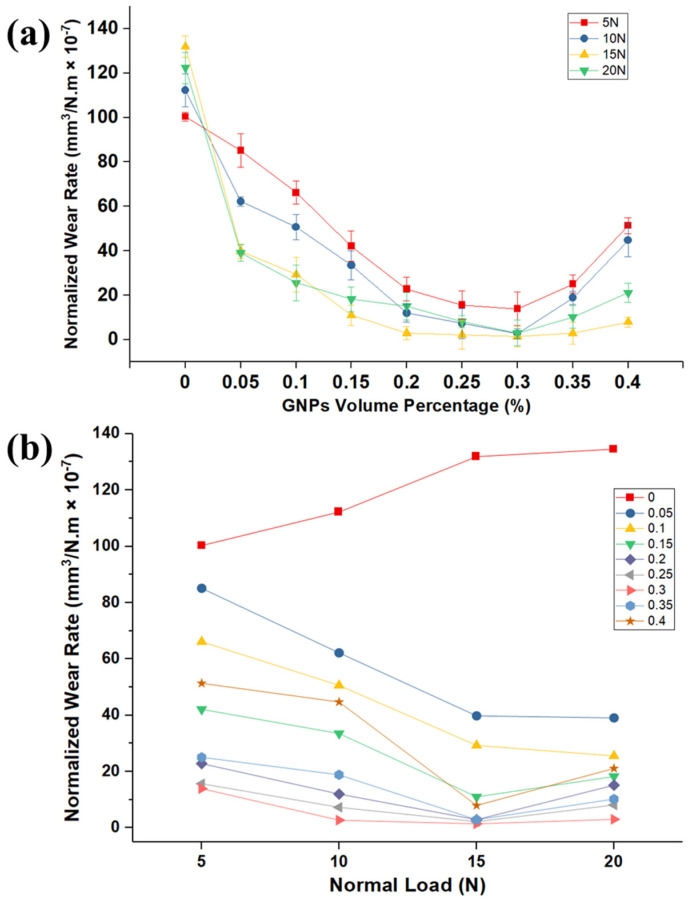
(**a**) The variation of wear rate in different volume percentage of GNPs at several loads. (**b**) The variation of wear rate in different loads at several volume percentages of GNPs.

The results of the COF and viscosity can be formulated in the form of well-known Stribeck curve [44], in an attempt to develop generalized observations as shown in Figure 4c. The parameters in the *x*-axis, P, η, and ω, are the average contact pressure, viscosity, and rotating speed, respectively, as shown in Figure 4c. The Stribeck curve can explain the variation of COF due to viscosity (temperature rise and particle concentration), along with pressure in one graph. The variation of COF with the bearing parameters considered in Figure 4 are presented by using the data shown in Figure 2 and Figure 3. The coefficient of friction decreases as more GNPs are added in the nanolubricant, as well as contact pressure increases. As contact pressure increases, more asperities in the contact surface yield and interact to each other and suffer plastic deformation. Lower resistance to the applied tangential load is exhibited, causing the overall friction coefficient to drop in accordance with existing friction theories [45,46,47,48,49]. Consequently, GNPs, as an oil additive, significantly reduce the COF into the boundary lubrication regime, as shown on the far left of the Stribeck curve. The variation of the pin wear volumes rate with GNP volume fraction in the nanolubricants for different loads is shown Figure 5a. It is clear from this figure that the wear rate of samples tested by nanolubricants is significantly less than those tested by neat oil at different normal loads, and GNPs are influential to improve the lubrication performance. Furthermore, this graph demonstrates that there is significant reduction of the pin wear volume rate associated with the increasing GNPs volume fraction for various applied loads. Thus, it is apparent from wear volume rate data that the nanolubricants with a high number of particles have the lower wear volumes rate.

It is generally accepted that the larger wear rate of neat oil can result from the high real contact area of the rubbing surfaces. As mentioned earlier, when GNPs were added to the base oil, they fill up the micro- and nano-gaps of the rubbing surfaces and form a lubricant tribolayer, which can smooth the surfaces and protect the surfaces. As it is not generally agreed that it avoids direct contact of the two mating surfaces and consequently reduces the wear. The influence of the more GNPs volume fraction to lower wear rate is far greater than the low volume fraction of GNPs. For example, by examining neat oil, the presence of the 0.1 and 0.3 vol.% of GNPs at 15 N applied load lowers the wear volume by 78% and 99% improvement, respectively, when compared to the neat oil.

Turning now to the wear-rate results, evidence on the curve of wear rate versus GNPs volume percentage is U-shaped. Consequently, the single most striking observation to the U-shaped graph was the presence of an optimum concentration of GNPs, where there is lower wear rate. For canola oil, the optimum concentration of GNPs as an additive in oil is 0.3 vol.%. It is important to highlight the fact that, by adding more GNPs in the oil beyond this optimum concentration, an increase in the wear rate will occur. As a result, the wear rate will go up beyond the optimum point of the curves (0.30 vol.%). There are several possible explanations for this result. When the GNPs concentration is above the optimum concentration, this results in the excessive additive in the base oil, leading to decrease in the load bearing capacity due to the formation of lumps and agglomeration of GNPs in the interface, which will result in a negative effect to protection of contact surfaces and higher wear rate. Hence, the findings of this study suggest that the reason for increasing of the wear rate after an optimum concentration is that the GNPs can easily aggregate in the nanolubricants during the rubbing the two surfaces, resulting in the increase of the wear rate. Aggregated GNPs are considered to act highly as third-body abrasive particles when sliding and can damage the pin surface by plastic deformation and ploughing, resulting in the high wear rate. Moreover, as put forward by previous research [17], the evidence leads to GNPs piling up between friction pairs due to excessive concentration of GNPs in oil and, accordingly, blocking the formation of lubricating film between mating surfaces; the lubricating film then becomes much more discontinuous, even causing a dry friction that can tend to increase the wear rate. A limitation of this study is the evidence for our hypotheses that are clarified. On the contrary, a GNP concentration less than 0.30 vol.% is not sufficient to cover and protect the majority of the surface and reduce the real contact area between the mating surface to reduce the wear rate. Therefore, nanolubricants with a lower concentration than 0.30 vol.% have higher wear rate (Figure 5a). Further explanation describes that particle additive concentrations below the optimum concentration result in insufficient load carrying capacity.

Figure 5b depicts the variation of wear rate of pins at different loads for several volume percentages of GNPs. By increasing the load, the wear rate of aluminum in presence of nanolubricants decreases, while the wear rate of neat oil increases. The plot also shows higher wear rate at higher normal load. 

#### 3.1.2. Characterization of Worn Surfaces 

**Figure 6:**
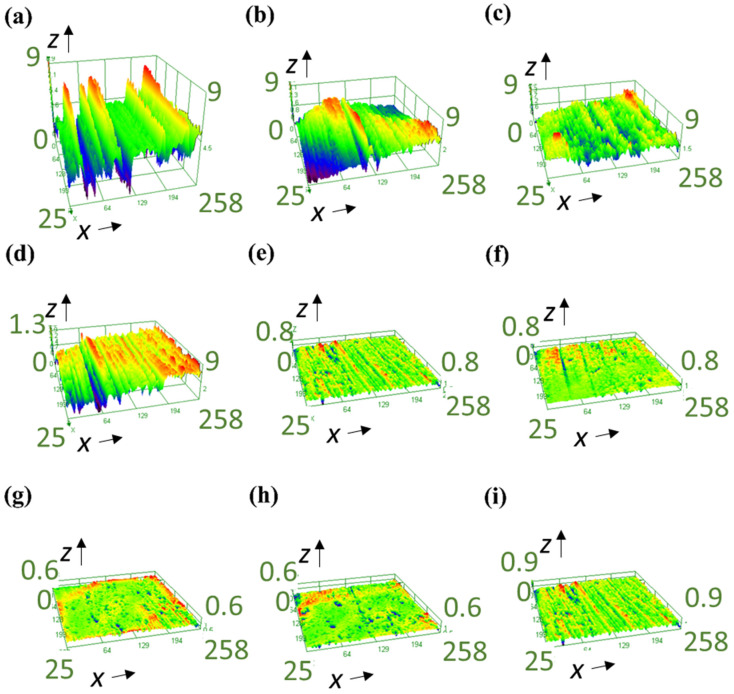
The 3D optical profilometer images of worn surface of the pin at 20 N for the (**a**) neat oil, (**b**) 0.05 vol.% nanolubricant, (**c**) 0.10 vol.% nanolubricant, (**d**) 0.15 vol.% nanolubricant, (**e**) 0.20 vol.% nanolubricant, (**f**) 0.25 vol.% nanolubricant, (**g**) 0.30 vol.% nanolubricant, (**h**) 0.35 vol.% nanolubricant, and (**i**) 0.40 vol.% nanolubricant.

To further evaluate and understand the influence of the GNPs volume fraction on the wear, a 3D optical profilometer was employed to analyze the topography of worn surface and measure surface roughness parameters. Figure 6 is a representative of three-dimensional worn surfaces of pins recorded by the optical profilometer at 20 N loads. In line with the wear-rate results (Figure 5), the surface analysis shows that the worn surfaces in presence of GNPs are much smoother than neat oil, as is expected. Comparing Figure 6a with other worn surfaces, the worn surface lubricated by the neat oil shows many thick and deep grooves, while shallower and narrower grooves appeared on the worn surfaces lubricated by nanolubricants, and the worn surfaces are comparably smoother for higher GNPs concentration. A similar conclusion as that of wear rate results was reached by analyzing topography of worn surfaces: Graphene can provide better finish on the worn surface.

**Figure 7:**
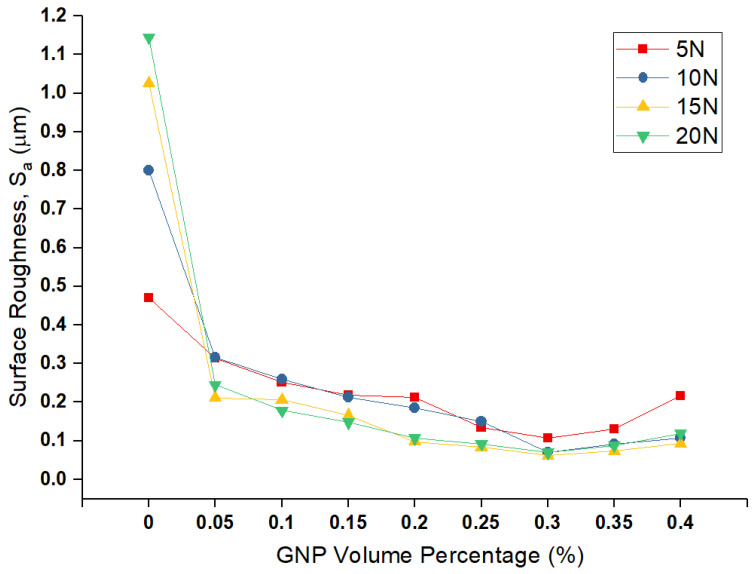
Surface roughness of worn surfaces of pins in several GNPs at different loads.

The *S*_a_ values of the worn pin surfaces for various nanolubricants at different normal load are depicted in Figure 7, which shows a relationship between the surface roughness and volume fraction of GNPs. By comparing the roughness value of neat-oil tested sample with nanolubricants, it is obvious that nanolubricants are effective to enhance the wear resistance, due to less damage and failure on the surface by having low surface roughness values. Moreover, the roughness value of the worn surface decreases by increasing the volume percentage of GNPs in nanolubricants up to 0.30 vol.%, with the same trend of wear rate. Hence, amongst the nanolubricants, the lowest roughness belongs to 0.30 vol.% nanolubricants, as is expected from wear data from Figure 5. Therefore, it can confirm the claim that GNPs can fill up the valleys of asperities, to smooth the surfaces, and reduce the surface interlocking and shear stresses between two surfaces, to reduce the wear. Besides, nanolubricant with a higher GNPs volume percentage can fill up more gaps, cover more area of surfaces, and, hence, provide more protection on the surfaces. Therefore, the surface finish is smoother for nanolubricants with more GNPs. On the other hand, adding GNPs more than 0.30 vol.% has a negative effect, as the surface roughness increases due to agglomerated GNPs, which behave like a third-body abrasive particle due to their plate-like geometry, which damages the pin surface by plastic deformation and ploughing and can cause an increase in the wear volume rate and, consequently, rougher surfaces. 

**Figure 8:**
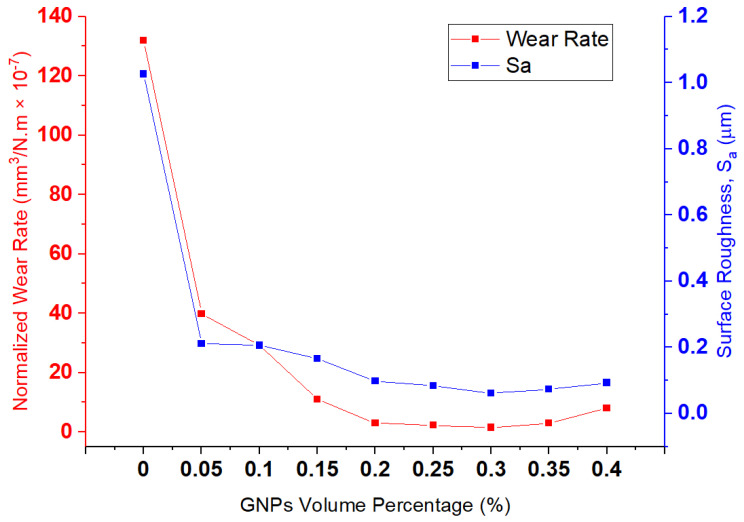
The relationship between wear rate, surface roughness (Sa), and GNPs concentration at 15 N.

Figure 8 shows the relationship between wear rate and surface roughness at 15 N. A direct relationship between *S*_a_ and wear rate, and also a sharp reduction in wear rate and *S*_a_ by adding 0.5 wt.% graphene, can be observed. Moreover, the surface-roughness increment from 0.35 wt.% to 0.40 vol.% graphene is in line with the increasing wear rate. Therefore, all samples are corresponding to a direct relationship between wear rate and surface roughness.

**Figure 9:**
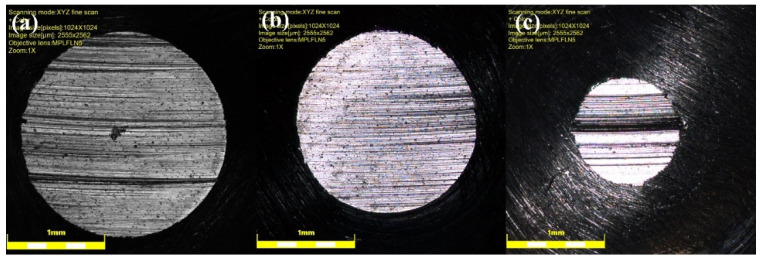
Wear-scar diameter of pins at 15 N for samples tested with (**a**) neat oil, (**b**) 0.10 vol.% nanolubricant, and (**c**) 0.30 vol.% nanolubricant.

**Figure 10:**
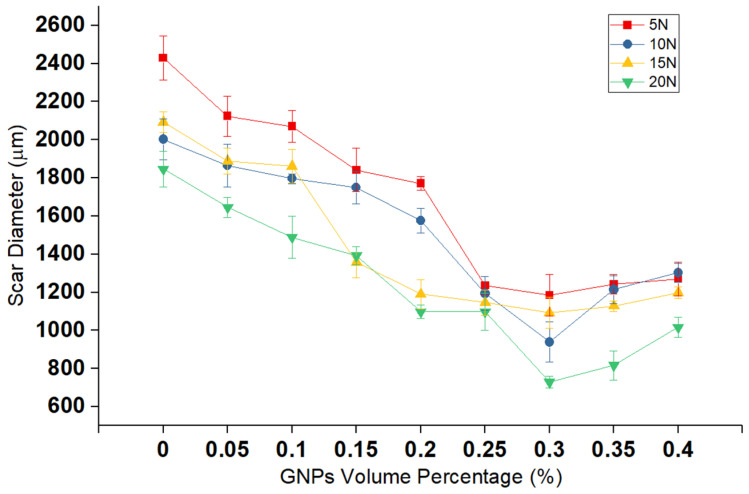
Correlation of wear-scar diameter pin with graphene volume percentage of nanolubricant at different applied loads.

After each test, the worn surface is flat, and each worn pin has a unique wear-scar diameter that relates to the wear rate of samples. Figure 9 depicts the scar diameter of the pin at 15 N applied load for neat oil and nanolubricants. As shown in Figure 9, the diameter of the pin is larger for the sample tested with neat oil (Figure 9a), in comparison with nanolubricant (Figure 9b,c), as is expected because of the higher wear rate for neat oil sample. Figure 10 exhibits the correlation between scar diameter and GNPs volume percentage at different loads. The trend of wear scar diameter versus volume fraction of GNPs is the same as that of wear rate versus surface roughness, as expected.

**Figure 11:**
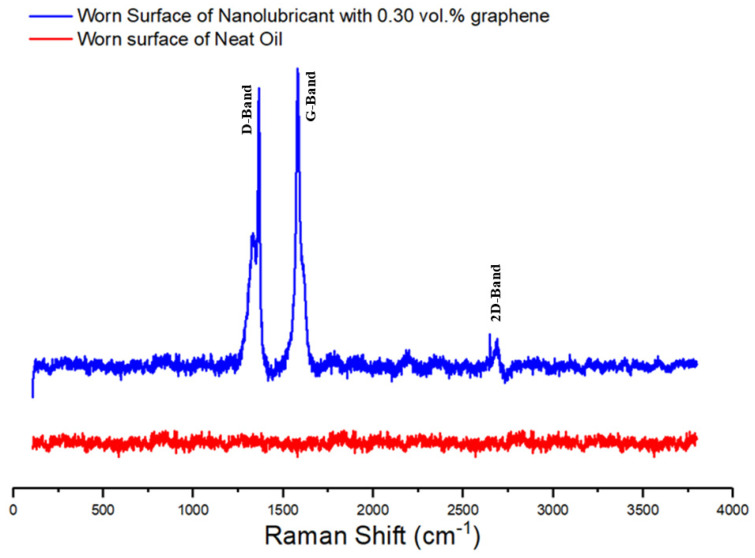
The Raman spectrum of the worn surface of nanolubricants with neat oil and 0.30 vol.% nanolubricants.

To have an understanding of the chemistry of surfaces, the full Raman spectra of worn surface of sample lubricated by neat oil and nanolubricant with 0.30 vol.% GNPs is shown in Figure 11. It is apparent from Raman spectra on the wear surface lubricated by the neat oil that the aluminum pin surface proves no existence of any type of carbonaceous materials, because of no existence of D-, G-, and 2D band in the Raman spectra. In contrast to this finding, however, evidence of carbonous materials was detected on the worn surface lubricated with the nanolubricant, due to the exhibition of strong D- and G-band in the Raman spectrum. This appears to be a case of partial covering of the worn surface with carbonaceous materials. Moreover, it is worth discussing these interesting facts that a disordered structure appears to be well supported by the presence of the D-peak in the Raman spectra. The intensity of the D peak after the tribology test increased significantly. The results of this study explain the occurrence of these phenomena. The I_D_/I_G_ ratio is equal to 0.96 after wear test (Figure 11), whereas the ratio was equal to 0.72 before the tests. This shows that the disorder in the ball milled GNPs crystallite structure increased slightly. The ratio of the D- and G-band intensities (ID/IG) is inversely proportional to the in-plane crystallite size L_a_ [50]. The in-plane crystallite size (L_a_) from the Raman spectra taken before and after the wear tests (Figure 1b and Figure 11) is calculated by using the general equation L_a_ = (2.4 × 10^−10^) λ^4^_laser_ (I_D_/I_G_)^−1^, where λ_laser_ is the wavelength of the laser light in nm unit [51]. The crystallite sizes obtained at different spots, using this equation, are given in Table 2. The in-plane crystallite size decreased significantly after the wear tests, as shown in Table 2. As a result, the analysis result verifies the deposition of a carbonaceous film on the worn surface during the wear process, and the wear track surface is almost covered by the carbonaceous film after the tribological test. Consequently, the addition of GNPs into the oil clearly gives a positive effect on friction and wear properties.

**Figure 12:**
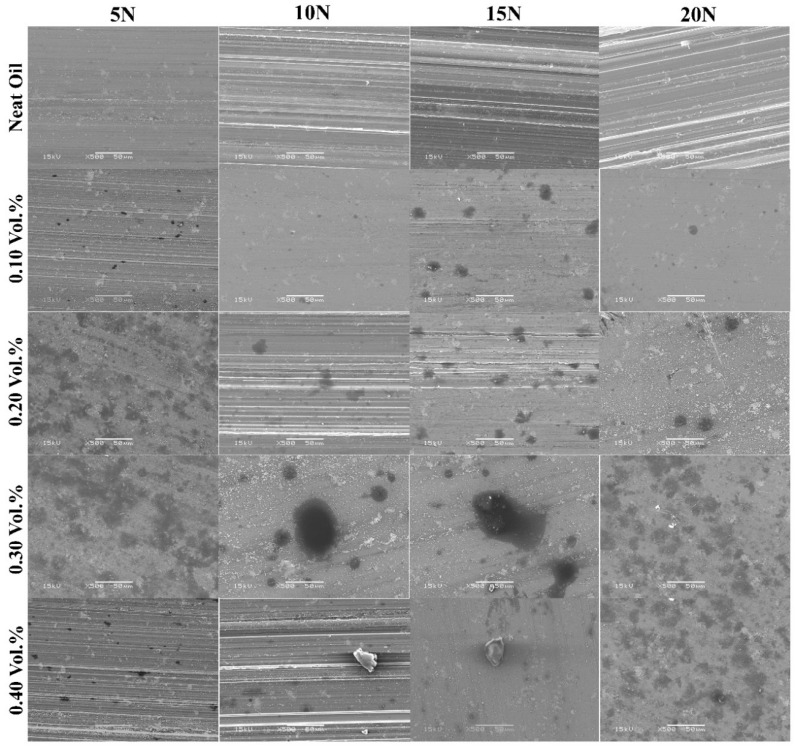
Scanning electron micrographs of worn pin surfaces for various nanolubricant at different loads.

The evidence of reducing the COF and wear rate in the presence of GNPs in nanolubricant can be proved by the results of SEM and EDX. Figure 12 shows scanning electron micrographs of worn pin surfaces for the particulate nanolubricants containing GNPs at different loads. It is obvious that the surface of samples tested by neat oil is rougher with many thick and deep crack. It is the result of having no protective layer deposited to shield the surface from more damage and abrasive wear, as compared to the worn surface of neat oil (first row) with nanolubricants (rows 2–5 in Figure 15). The pin surface used in the neat oil is severely abraded, having a high *S*_a_ value (Figure 7), which is significantly rougher than nanolubricant tests. These are in contradiction with the worn surface of samples tested by nanolubricants, where carbonaceous film deposition (black spots) appears on the worn surfaces of the aluminum pin as lubricant tribolayer, unevenly distributed over the surface. A possible explanation for these results may be due to the GNPs particles or agglomerated particles, as a lubricant tribolayer can be rolled off, and they are likely to act as nano-rolling and nano-bearing elements, which results in low friction coefficients and wear during the test. A carbonaceous tribolayer is formed on the worn surface of nanolubricants, as shown in Figure 12. These findings are consistent with the result of wear rate (Figure 5) that demonstrate the lower COF and wear rate for samples tested by nanolubricants in compression with samples tested by neat oil. There is a speculation to explain this result, but it might be related to that the particles can penetrate the inter-asperity valleys and fill them to create a smoother final worn surface and protect the surface from more damage.

To understand the effect of GNPs concentration for each applied load, it can be revealed that the surface becomes smoother and the roughness value of the worn surface decreases from top to bottom in Figure 12, at a higher volume fraction of GNPs. The smoothest surface was observed in 0.30 vol.% nanolubricant. In fact, in this trial the presence of the 0.30 vol.% GNP particles lowered the COF by 82%, 74%, 73%, and 76%, the pin wear volume by 86%, 98%, 99%, and 98%, and the surface roughness by 77%, 91%, 94%, and 94% at 5, 10, 15, and 20 N, respectively. Therefore, from the short discussion above, key findings emerge that adding 0.30 vol.% is the optimum concentration of GNPs that can cover and form a good uniform tribolayer on the worn surface to preserve the pin surface from wear and damage. On the contrary, a comparison of the worn surface of samples tested by 0.30 and 0.40 vol.% GNPs reveals that increasing the concentration of GNPs particles more than 0.3 vol.% results in more wear in aluminum pin, due to agglomeration of GNPs particles, which act as third-party abrasive particles, as more scratches were observed on the worn surfaces of samples tested by 0.40 vol.% nanolubricant, as discussed earlier. 

A further important implication is that more of the area of the worn surface of the sample tested by 0.3 vol.% nanolubricant is covered by lubricant GNPs tribolayer than the area of surface covered by 0.1 vol.% nanolubricant, for example, as shown in first column of Figure 12, at 5 N; thereby, it reaches the conclusion that there is less real contact area between pin and disk for samples tested by 0.3 vol.% nanolubricant, and it can protect more asperities. This was included to verify that lower COF of samples tested by 0.30 vol.% nanolubricant than samples tested by 0.1 vol.% nanolubricant. Generally, the COF reduces by increasing the solid lubricant GNPs additive into nanolubricants (Figure 2a). On this basis, it can be concluded that the nanoparticles usually form a thin transfer layer on the surface of the tribocontacts that can support partial hydrodynamic forces; therefore, the surface-to-surface contact of the asperities is reduced, resulting in less friction, wear, and surface damage. The present findings confirm that adding GNP particles to oil can be effective. 

To advance the understanding of wear behavior, the characterization of the worn surface was made. Third column of Figure 12 compares the worn surface of aluminum pins at 15 N for neat oil and nanolubricant with 0.1 and 0.3 vol.% of GNPs. Hence, there is no evidence for the formation of any tribolayer on the worn surface of sample tested by neat oil (Figure 12), while the SEM image provides evidence that the formation of several black spots on the worn surfaces are the nano-lubricant tribolayer, as shown in Figure 12. This may be the reason why the real contact area between two surfaces was reduced for nanolubricants. As a consequence of the reduction in the real contact area, wear rate decreases for samples tested by nanolubricants, in comparison with neat oil. The analyses examined the impact of GNPs on wear rate where the larger area of the worn surface is deposited with carbonaceous film by increasing the volume fraction of GNPs, and thus greatly reducing the roughness of the surface and direct surface-to-surface contact, and giving better lubrication properties. It is important to note that the present evidence relies on less contact between asperities, as less failure and deformation occur and, hence, a lower wear rate is expected. By comparing the worn surface of the samples tested by the nanolubricants with 0.1 and 0.3 vol.% of GNPs, it is obvious that more of the surface area of the aluminum pins tested with 0.3 vol.% nanolubricant was covered by a carbonaceous tribolayer, compared to that of the nanolubricant with 0.1 vol.% of GNPs. Therefore, there is less real contact area between pin and disk for 0.3 vol.% GNPs, and so the wear rate is less than 0.1 vol.% nanolubricant. 

**Figure 13:**
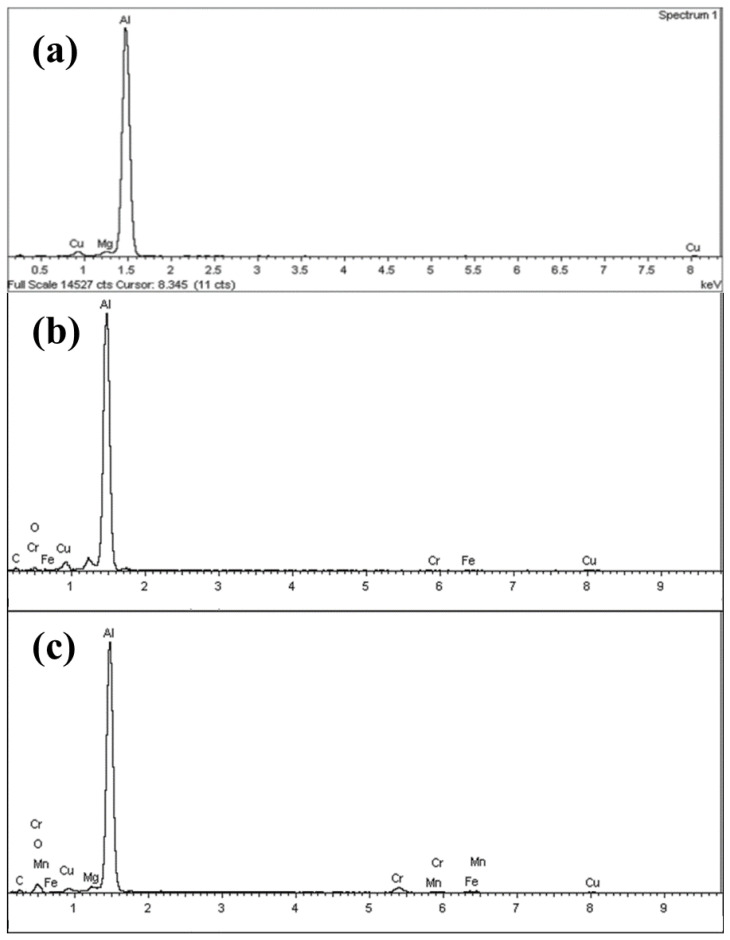
(**a**) Spectrum of unworn aluminum samples before the test. EDX spectrum of worn surfaces of aluminum pin samples for neat oil after the test at (**b**) 10 N and (**c**) 20 N.

To investigate the chemical composition of worn surfaces, EDX was employed. Figure 13a presents the EDX spectrum of pin surfaces before the wear test. As one expects, a majority of the unworn surfaces are made of aluminum with some minor trace of the copper and magnesium because the pin-sample surfaces studied in this work are the 2024 aluminum pin. Meanwhile, Figure 13b,c is EDX results of the worn samples under a neat oil and nanolubricants that demonstrate a majority of worn surfaces are composed of aluminum and a trace of iron and chromium on the surface and inside the wear grooves. 

**Figure 14:**
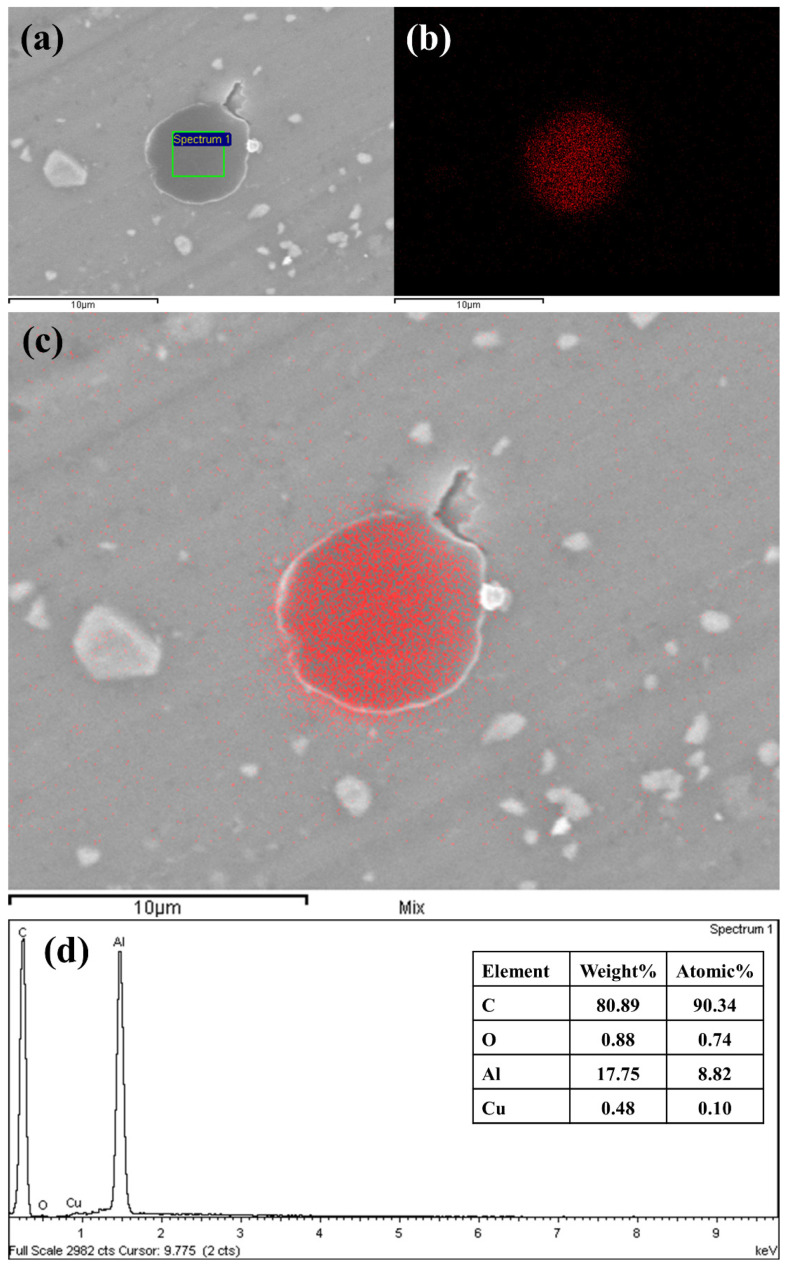
SEM image and elemental mapping for the surfaces tested with 0.30 vol.% nanolubricant at 20 N (**a**) and its material composition, using EDX (**b**–**d**).

The EDX analysis for a sample surface lubricated with 0.3wt.% of GNPs at 20 N load is depicted in Figure 14. Strong evidence of traces of the element carbon on the surfaces was found from the EDX results when the surface was exposed to nanolubricants during the test. This suggests that GNPs play a significant role in acting upon the contact surfaces where the high concentration of carbon on the surface now provides evidence that the GNPs are still largely intact on the surface. Together, these results provide important insights that the lubricant tribolayer is deposited on the worn surfaces for tested samples with nanolubricant containing GNPs. This is in contrast to the EDX of the worn surface, which was tested by neat oil with no carbonaceous film, as it has no signs of carbon. Figure 14a–c shows the carbon element mapping of the worn surface. Generally, the darker areas in the SEM images belong to higher concentrations of carbon, signifying that GNPs may be transferred and deposited in these dark areas of worn surface and form a tribolayer. The result of this analysis indicates a distribution of nanoparticles transferring and sticking to the worn surface, inside and outside of the wear grooves that the notable improvements in friction and wear rate are achieved. The same opinions represent other tested samples where the carbon element trace appears on the SEM and EDX results and exhibits a random carbon distribution on the worn surfaces. Using EDX, the average weight fraction of carbon detected on the surfaces tested by nanolubricants was measured to be 12–95%, depending on the volume fraction of GNPs into nanolubricants. This result ties well with previous results, wherein the GNPs establish a physical deposition tribolayer on the worn surface and avoid surfaces from direct contact-to-contact and, consequently, decrease the COF and the wear rate of the materials by showing superior anti-wear properties of nanolubricants.

#### 3.1.3. Wear Mechanism

The main objective of the paper is to explain the wear mechanisms. Several mechanisms have been proposed for various tribological enhancements, using different types of oil additives. Our results are consistent with what has been found in a prior study by Lin et al. [16] that proves that enhancing the load-carrying capacity can improve the wear resistance of graphene nanolubricant and modify the surface. The SEM and EDX results thus obtained are compatible with our study that validates the probability of the formation of a thin laminated structure on the worn surface, due to ability of the GNPs to easily penetrate between the contact surfaces. Furthermore, the improvement in anti-wear ability can be attributed to GNPs’ intrinsic structure and lubrication nature. A different approach to the wear test limitation is solved by atomic force microscopy (AFM). AFM based friction studies of graphene substrates have been instrumental in explaining various possible mechanisms [31,52]. Electron–phonon coupling [53], puckering effect [32], and interplay of surface attractive forces [54] in graphene play a vital role in improving tribological properties.

**Figure 15:**
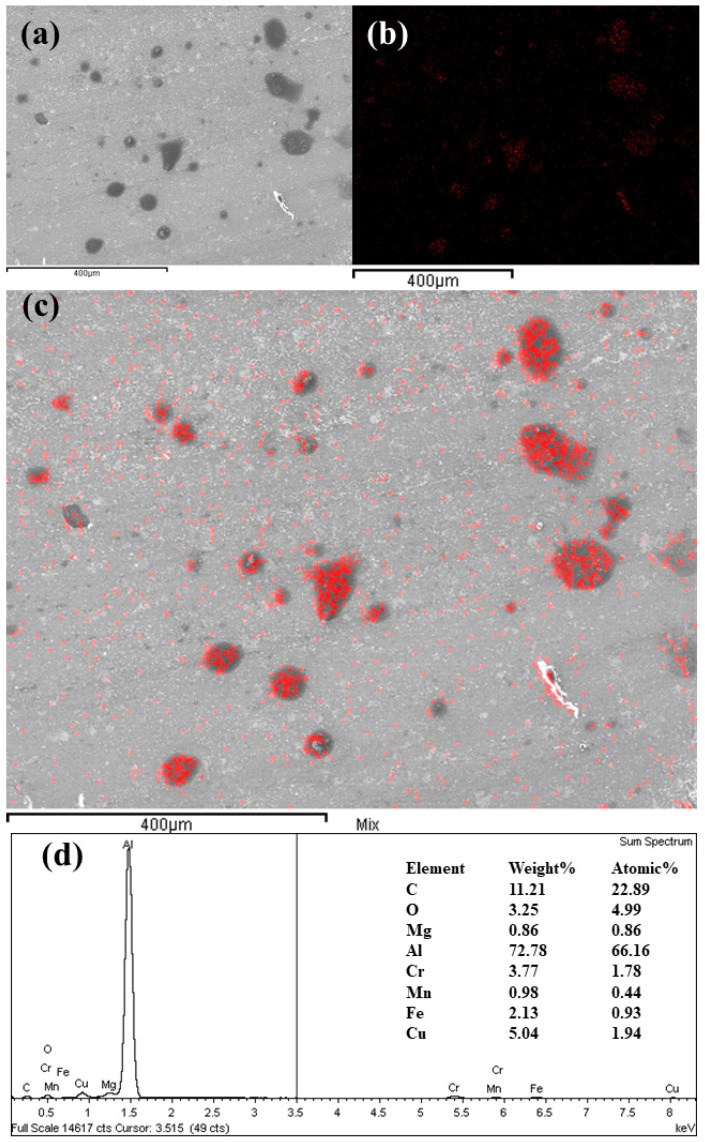
SEM image and elemental mapping for the surfaces tested with 0.20 vol.% nanolubricant at 20 N (**a**) and its material composition, using EDX (**b**–**d**).

Based on the experimental observations, it was concluded that the occurrence of several morphological transformations and deposition of GNPs simultaneously or subsequently can play a key role on tribological behavior. The large size variation exists when GNPs are dispersed in the based oil [55]. Therefore, the nanolubricants should be a poly-dispersed GNPs–lubricants mixture [56]. Small GNPs could easily penetrate in the valleys and prevent the deepening of the same. Large GNPs could provide coating effect by sliding, buckling, bending, or by turning into semi tubes as the shear forces act on them, as proven by Rasheed [57]. From these standpoints, it can be introduced a possible confirmation in GNPs’ effect, where GNPs can slide between the mating contacts, especially during the boundary lubrication, thereby helping the formation of a protective tribolayer that could be principally created due to the layer structure of GNPs. The aforementioned, SEM images illustrate that the GNPs are penetrated and deposited in valleys and ridges on the grooves of worn surfaces, and EDX analysis prove the presence of carbon deposition in wear tracks, as shown in Figure 15.

**Figure 16:**
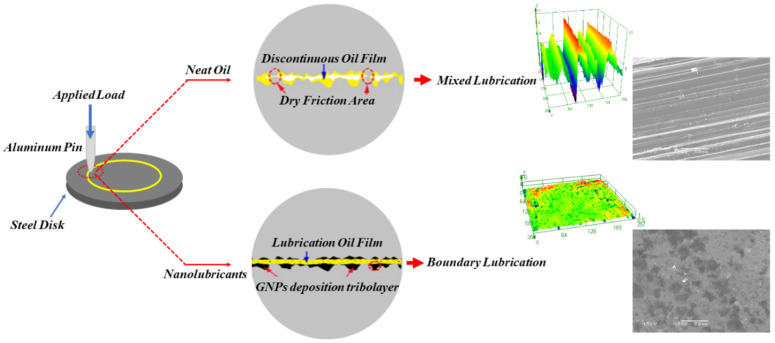
Schematic of the lubricating models of the neat oil and the nanolubricants.

This paper proposes the lubrication models of the neat oil and the nanolubricant. Figure 16 illustrates the schematic of the lubrication models. In the case of lubrication with the neat oil, two contact surfaces make contact on each other and produce scratches and debris because of the larger frictional force. The debris get trapped between sliding surfaces, making the contact surface extremely rough. Once the surface roughness is greater than the oil-film thickness, the dry contact condition will happen, and consequently, wide and deep grooves and furrows are shaped on the worn surface. Contrary to the neat oil lubrication model, the GNPs in nanolubricants can penetrate and slide the mating surfaces, and they gradually deposit and accumulate on the worn surface during the wear test and the surface becomes smoother. The primary cause of the flat and smooth surface is a consequence of the filling up of grooves by GNPs. The findings at least indicate that the surface roughness of samples tested by nanolubricants significantly drops, compared with that lubricated by the neat oil. This can raise interest about lubricant tribolayer confirmed by SEM and Raman, as the deposited GNPs produce a deposition lubricant tribolayer that can cover the worn surface. Under certain observations, this can result in fewer abrasive particles, making it easier to maintain the smooth contact surfaces and reduce the wear volume. 

In addition, this phenomenon can be explained as the lubrication regime transition [17]. where *h* is the thickness of the lubrication film, and *R_q_* is the roughness of the surface. As explained earlier, the lubricant tribolayer can reduce the roughness of the worn surface, resulting in an increase in *h*/*R_q_*; thus, the lubrication goes up into the BL regime. At the higher concentration of GNPs, i.e., more than 0.30 vol.%, GNPs will accumulate between contact surfaces and block the oil film from lubricating the surfaces. As a result, the thickness of lubrication film decreases, and then *h/R_q_* drops. Consequently, the lubrication enters a transition region between boundary and dry lubrication regime again. To designate the effect of load on the lubrication regime, elastic deformation of the GNPs takes place at a higher load, and it will reduce the buffering friction. Since the thickness of GNPs tribolayer in the present case is in nanosized range, this can form a nanobearing between moving surfaces. This may result in sliding when the excess load is applied. Hence, added additive to the base oil can act as a mechanical reinforcing element during friction and can, therefore, strengthen the load-carrying capacity of the nanolubricants. 

As the GNPs are the absorbent of lubricants, it can be suggested that the graphene can absorb base lubricant, which thickens the lubricant film and prevents the friction pairs from direct contact. As a result, the lubrication regime in the nanolubricants has transferred to a better lubrication regime, which tends to be a tremendous improvement in COF reduction and wear-resistance behavior [58]. In summary, two important roles of GNPs are explained: Firstly, the graphene nanosheets enter the contact zone and roll between the two contact surfaces. Secondly, during the sliding, the high contact pressure creates stressed zones of traction/compression that then lead to the formation of a thin physical tribolayer on the contact surfaces. The physical tribolayer could not only bear the load but also prevent the two metal surface from making direct contact. Therefore, the anti-wear ability of the nanolubricant was improved, and the friction coefficient and wear were decreased significantly.

## 4. Conclusions

The main conclusion that can be drawn is that GNPs are effective to enhance the lubrication performance of canola oil as a lubricant additive. The findings of this study can be understood as follows: The COF decreased for nanolubricants where the COF improved 14%, 27%, 52%, and 33% by adding just 0.05 vol.% GNPs at 5, 10, 15, and 20 N, respectively. When comparing the results to COF at different concentrations of GNPs, it must be pointed out that increasing the concentration of GNPs can decrease the COF, where the COF decreases by 83%, 79%, 84%, and 83% for the GNPs concentrations of 0.3 vol.% at 5, 10, 15 and 20 N, respectively. This conclusion follows from the fact that the steady-state temperature is also lower for nanolubricants, and this could be the result of the higher thermal performance of nanolubricants and/or the lower friction of the nanolubricants. There is an optimum concentration of GNPs where the wear rate is in the lowest value. By increasing the load, the wear rate of aluminum in the presence of nanolubricants decreases, while the wear rate of neat oil increases. Results provide a basis for the viscosity of nanolubricants where no significant change occur in the viscosity by adding the various GNPs concentrations. It is to be noted that GNPs present higher barrier properties in humid environments. The addition of GNP decreases the maximum water content absorbed and the diffusion coefficient. However, this is not too large, due to the weak interface with water or other aqueous elements that may have contaminated the lubricants.

Importantly, our results provide evidence for the role of GNPs in the nanolubricants, as well as the wear mechanism, by examining the worn surface. The worn surface and surface-roughness value show that lubricated surfaces with nanolubricants are smoother, since narrower and shallower grooves exist. There is a direct correlation between wear rate and surface roughness. Wear-scar diameters for each sample were measured, and it was shown that they decreased by increasing the volume percentage of GNPs up to 0.30 vol.%. Then the wear-scar diameters increased by increasing the concentration above 0.30 vol.%. Broadly translated, the EDX results indicate that aluminum is the dominant element of the worn surface with a trace of iron and chromium on the worn surface, while the worn surfaces of samples tested by nanolubricants exhibit the presence of carbon element on the worn surface, due to the formation of tribolayer. Collectively, our results appear consistent with Raman spectroscopy data that the wear track of samples tested by nanolubricants exhibits strong D- and G-band in the Raman spectrum that confirm worn surface covered with GNPs tribolayer. This allows the conclusion that the deposition of carbonaceous tribolayer on the worn surface during the wear process. Based on the results and characterization of worn surfaces, this may be considered a promising aspect of different possible enhancing mechanisms that the reduction of the real area of contact by forming lubricant carbonaceous tribolayer was proposed as the dominant mechanism in this work. The proposed mechanism is compatible with the friction and wear experimental data. 

## Figures and Tables

**Table 1:** Pin-on-disk test parameters.

Parameters	Value
**Loads**	5, 10, 15, and 20 N
**Sliding speed**	25 mm/s
**Sliding distance**	2000 m
**Temperature**	23 °C

**Table 2:** In-plane crystallite size, L_a_, of GNPs calculated by using the Raman spectrum before and after the wear tests.

	I_G_/I_D_	L_a_ (nm)
**As-received GNPs**	1.6	63
**Ball-milled GNPs**	1.4	54
**After Wear Test**	1.04	40

## Data Availability

Data available on request due to restrictions eg privacy or ethical. The data presented in this study are available on request from the corresponding author. The data are not publicly available due to propriority materials, equipment, and fabrication process being studied.
